# Early Diagnosis of Bloodstream Infections Using Serum Metabolomic Analysis

**DOI:** 10.3390/metabo14120685

**Published:** 2024-12-06

**Authors:** Shuang Han, Ruihua Li, Hao Wang, Lin Wang, Yiming Gao, Yaolin Wen, Tianyang Gong, Shiyu Ruan, Hui Li, Peng Gao

**Affiliations:** 1 Department of Clinical Laboratory, The Second Affiliated Hospital of Dalian Medical University, Dalian 116023, China; hanshuang0967@dmu.edu.cn (S.H.); lrhzyp2006@dmu.edu.cn (R.L.); 2 School of statistics, Dongbei University of Finance and Economics, Dalian 116025, China; hao.wang@dufe.edu.cn (H.W.); 2023000155@stumail.dufe.edu.cn (L.W.); 2023121149@stumail.dufe.edu.cn (Y.G.); 2023121071@stumail.dufe.edu.cn (Y.W.); 19950032@dufe.edu.cn (T.G.); 20100039@dufe.edu.cn (S.R.)

**Keywords:** metabolomics, bloodstream infection, bacteria, fungi, extended-spectrum β-lactamase

## Abstract

Background: Bloodstream infections (BSIs) pose a great challenge to treating patients, especially those with underlying diseases, such as immunodeficiency diseases. Early diagnosis helps to direct precise empirical antibiotic administration and proper clinical management. This study carried out a serum metabolomic analysis using blood specimens sampled from patients with a suspected infection whose routine culture results were later demonstrated to be positive. Methods: A liquid chromatograph-mass spectrometry-based metabolomic analysis was carried out to profile the BSI serum samples. The serum metabolomics data could be used to successfully differentiate BSIs from non-BSIs. Results: The major classes of the isolated pathogens (e.g., Gram-positive and Gram-negative bacteria) could be differentiated using our optimized statistical algorithms. In addition, by using different machine-learning algorithms, the isolated pathogens could also be classified at the species levels (e.g., *Escherichia coli* and *Klebsiella pneumoniae*) or according to their specific antibiotic-resistant phenotypes (e.g., extended-spectrum β-lactamase-producing and non-producing phenotypes) if needed. Conclusions: This study provides an early diagnosis method that could be an alternative to the traditional time-consuming culture process to identify BSIs. Moreover, this metabolomics strategy was less affected by several risk factors (e.g., antibiotics administration) that could produce false culture results.

## 1. Introduction

Bloodstream infections (BSIs), which are caused by the invasion of pathogenic microorganisms into the circulatory system, are a critical public health issue [1]. If proper clinical management is not applied in a timely and effective manner, BSIs will often progress rapidly and result in severe systemic illnesses, such as sepsis and septic shock [2]. For severe sepsis and septic shock patients, their survival rates decreased by an average of 7.6% for each hour that effective antibiotic administration is delayed [3]. The incidence of BSIs varies across different regions and populations [4,5]. A study carried out in Finland estimated that the incidence of BSIs is 0.1% to 0.2%, whereas the incidence in Switzerland is about 2.2% [5]. Patients admitted to the intensive care unit (ICU) are prone to suffering from BSIs and have a higher mortality rate [6,7]. 

Currently, blood culturing (BC) is the most recommended method for diagnosing BSIs. However, to obtain a culture result requires at least 12 to 48 h [2,8]. Even the recently introduced method for the rapid identification of bacteria using matrix-assisted laser desorption ionization time-of-flight mass spectrometry (MALDI-TOF-MS) still requires pure cultures. The time-consuming culture procedure does not allow for an early diagnosis and immediate therapeutic interventions. Additionally, many strains that can be easily identified using traditional methods cannot be differentiated by means of MALDI-TOF-MS, e.g., differentiating *Shigella* spp. from *Escherichia coli* (*E. coli*) [9]. The long turnaround time of BC often necessitates the empirical use of broad-spectrum antibiotics for patients with suspected infections [2,10]. Even so, approximately 40% of empirically treated BSI cases required treatment adjustments after their BC results become available [11]. Moreover, to improve the culture-positive rate, all the approved guidelines recommend collecting blood samples before antibiotic administration. Unfortunately, this prerequisite is often not feasible in practice. It was reported that about 28% to 63% of fever patients had received or were receiving antibiotic treatment when their blood was sampled [12,13,14], leading to no viable organisms in the samples and subsequent false-negative BC results [15]. Moreover, false-negative BC results could also result from the presence of non-cultivatable or fastidious bacteria [16,17]. Since the advent of the polymerase chain reaction (PCR) technique, the relevant non-culture-based methods have thrived in clinics. The applicability of traditional PCR-based methods depends on the precise selection of targeted microbe-specific primers, whose efficacy is usually unpredictable [18]. Metagenomic next-generation sequencing (mNGS) is, in theory, regarded as a universal unbiased method for detecting microbes, but only a limited number of laboratories can provide mNGS services. Moreover, routine applications of mNGS can encounter many obstacles, such as high costs, long turnaround times, contamination, and difficulties in data interpretation [19]. Most importantly, a sufficient amount of the target bacteria is a prerequisite for a positive mNGS result.

The incidence of BSIs is increasing year over year [20,21,22]. The above-mentioned diagnostic limitations of BC underscore the need for an earlier and more precise diagnostic technique [10,23]. All the metabolites in a given system constitute the system’s metabolome. Metabolomics is a high-throughput technique for quantifying as many metabolites as possible in a single run [24,25]. It can identify the phenotype features of an organism, which reflect the genetic and environmental effects on an organism. When the patients suffer from BSIs, the living pathogens can release their metabolites into the patients’ blood. When the patients respond to BSIs, their serum metabolome will be altered. Therefore, the fluctuating metabolites are from both the host and the pathogens [26]. Thus, we used metabolomic analysis to profile serum metabolites. It was found that samples from BSI and non-BSI patients could be differentiated, and the two most commonly isolated species from the BSI patient samples could be clearly identified. Furthermore, bacteria with different antibiotic-resistant phenotypes could also be confirmed. These results demonstrate that serum metabolomic analysis is a valuable strategy for diagnosing BSIs and guiding early targeted antibiotic treatments.

## 2. Materials and Methods

### 2.1. Blood Sample Collection 

In total, 109 patients with positive BC results were enrolled in this study. The isolated strains included *E. coli* (*n* = 45), *K. pneumoniae* (*n* = 28), *Staphylococcus aureus* (*S. aureus*, *n* = 8), *Enterococcus faecalis* (*E. faecalis*, *n* = 3), *Enterococcus faecium* (*E. faecium*, *n* = 3), *Bacteroides* spp. (*n* = 5), and *Candida parapsilosis* (*C. parapsilosis*, *n* = 3). The remaining 14 strains were other *enterobacteriaceae* and non-fermenting bacteria (*Providencia rettgeri*, *n* = 3; *Klebsiella oxytoca*, *n* = 2; *Proteus mirabilis*, *n* = 1; *Acinetobacter baumannii*, *n* = 3; *Pseudomonas aeruginosa*, *n* = 3; *Stenotrophomonas maltophilia*, *n* = 1 and *Pantoea* spp. *n* = 1). For each patient, two whole-blood samples were collected when fever symptoms appeared. One sample was used for BC and the other for metabolomic analysis. For the controls, 117 whole-blood samples were collected from gender- and age-matched healthy individuals. The blood samples were collected into serum separation gel tubes with no anticoagulant. Each tube was centrifuged at 3500 rpm for 10 min, and the supernatant was transferred into a 1.5 mL centrifuge tube and stored at −80 °C until the subsequent metabolomic analysis was performed. 

### 2.2. Bacterial Identification and Antibiotic Susceptibility Testing (AST)

Pathogen identification and AST were performed using the VITEK 2 system (BioMérieux, Marcy-l’Étoile, France). For the isolated *E. coli* and *K. pneumoniae*, an extended-spectrum β-lactamase (ESBL)-producing test was conducted for each strain. Finally, 38 ESBL-positive (ESBLs(+)) and 33 ESBL-negative (ESBLs(−)) strains were identified. 

### 2.3. Sample Preparation for Metabolomic Analysis

Each sample was subjected to polar metabolites and non-polar lipids extraction for metabolomic analysis. For the hydrophilic polar metabolite extraction, every 150 μL aliquot of serum was mixed with 600 μL of methanol–acetonitrile (1/1, *v*/*v*). The mixture was vortexed for 120 s and then centrifuged at 15,000× *g* for 15 min at 4 °C. Subsequently, 600 μL of the supernatant was transferred into three individual centrifuge tubes with 200 μL of aliquot in each. The samples were dried in a low-temperature centrifugal concentrator (Labconco Corporation, Kansas, MO, USA) and stored hermetically at 4 °C until the analysis. For the non-targeted liquid chromatography–mass spectrometry (LC-MS) metabolomic analysis, the samples were dissolved in 100 μL of a 50% methanol–water solution (containing multiple isotope-labeled internal standards) [27,28]. A quality control (QC) sample was prepared by pooling a fixed volume of each patient’s extracted sample into one tube. The QC sample was repeatedly analyzed, and the corresponding data were used to evaluate each analysis method.

For the extraction of hydrophobic non-polar lipids, every 20 μL of thawed serum was mixed with 120 μL of methanol (containing isotope-labeled internal lipid standards of phosphatidylcholine(PC15:0/18:1(d7)), triglyceride (TG15:0/18:1(d7)/15:0), sphingomyelin (SM18:1(d9)), lysophosphatidylcholine (LPC18:1(d7)), phosphatidyl glycerol (PG15:0/18:1(d7)), cholersteryl ester (ChE18:1(d7)), Lysophosphatidylethanolamine (LPE18:1(d7)), and phosphatidylethanolamine (PE15:0/18:1(d7))). The mixture was vortexed for 180 s. Next, 360 μL of methyl tert-butyl ether and 100 μL of water were pipetted into each tube, and the mixture was vortexed for 10 min incubated at 4 °C for 10 min and then centrifuged at 15,000× *g* for 15 min. Each 300 μL of the upper layer (containing the lipid extract) was transferred into a 1.5 mL centrifuge tube and dried under vacuum in a centrifugal concentrator (Thermo Scientific) as mentioned above. The lipid extracts were then dissolved in 150 μL of an acetonitrile–isopropanol mixture (containing the SPLASH^®^ LIPIDOMIX mixture internal standard) for non-targeted lipidomic analysis [27,28]. The QC sample was prepared and run as mentioned above.

### 2.4. Metabolomic Analysis

The metabolomic analyses were conducted using an Ultimate™ 3000 ultra-high-performance liquid chromatography (UHPLC) system (Thermo Scientific, San Jose, CA, USA) coupled to a Q Exactive™ quadrupole Orbitrap MS (Thermo Scientific, San Jose, CA, USA). The first fraction of the hydrophilic extract was separated using an ACE C18-PFP column (Advanced Chromatography Technologies Ltd., Aberdeen, Scotland) and analyzed in positive electrospray ionization mode. The second fraction was analyzed using an Acquity HSS C18 column (Waters Corporation, Milford, PA, USA) and negative electrospray ionization mode. The third polar fraction underwent hydrophilic interaction chromatographic analysis using an Acquity BEH Amide column (1.7 μm, 2.1 × 100 mm^2^, Waters Corporation, Milford, PA, USA). The detection mode was set to negative electrospray ionization mode. The chromatographic separation mobile phase comprised a mixture of 10% acetonitrile (as the weaker eluent) and 50% acetonitrile (as the stronger eluent), combined with 10 mM ammonium acetate, which acted as a salt buffer to enhance the separation efficiency. The lipidomic analysis was operated in positive/negative polarity switching mode using an Accucore C30 core–shell column (Thermo Scientific, San Jose, CA, USA). Full-scan mass spectra were acquired at a resolution of 70,000 FWHM, along with the top 7 or 10 full-scan data-dependent MS/MS spectra using XCalibur software (Thermo Scientific, San Jose, CA, USA).

### 2.5. Metabolomic Data Processing

The full-scan and data-dependent MS2 metabolic profile data [27,28] were processed using Compound Discoverer software (Thermo Scientific, San Jose, CA, USA) for comprehensive component extraction. The hydrophilic metabolites were structurally annotated by comparing the acquired MS2 spectra against both a local proprietary iPhenome™ SMOL high-resolution MS/MS spectrum library, developed using authentic standards, and the mzCloud library (Thermo Scientific, San Jose, CA, USA). Additionally, the exact mass/charge (*m*/*z*) ratio of the MS1 spectra were searched against The Human Metabolome Database [1]. The corresponding retention time and high-resolution MS/MS spectra similarity were also utilized to aid the metabolite identification. The lipid identification results were reconfirmed according to the classification criteria proposed by the Metabolomics Standards Initiative (MSI) for lipidomic standardization [2].

### 2.6. Statistical Analysis

The metabolomic data were initially normalized as previously described [3]. Subsequently, the data were trimmed and log-transformed for further processing. For the separation between BSI and non-BSI cases, a principal component analysis (PCA) and orthogonal partial least squares discriminant analysis (OPLS-DA) were conducted using SIMCA software (Sartorius AG Umetrics, Goettingen, Germany). Univariate analyses, including independent sample t-tests and false discovery rate adjustments, were performed using the Benjamini–Hochberg method and ChemRICH chemical enrichment analysis as needed [4]. 

The data visualization was generated using a proprietary cloud computing platform based on R (IPOS; URL: http://82.157.20.231:3838/ipos/, accessed on 24 January 2024). The metabolic pathway enrichment analysis of the differentially expressed metabolites was performed using the online MetaboAnalyst tool [5]. The data analysis and feature (metabolite parameter) selection were performed using Python (Version 3.8) and R (Version 4.3).

## 3. Results

### 3.1. Differentation Between BSI and Non-BSI Cases 

**Figure 1:**
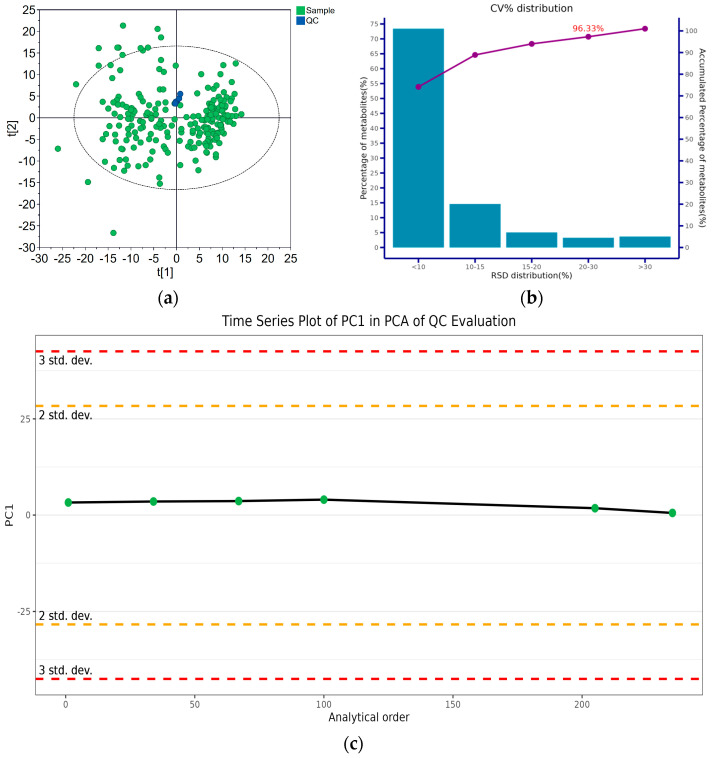
Evaluation of the metabolomic method stability for the first fraction of the hydrophilic extract analysis: (**a**) PCA score plot of the QC and real samples; (**b**) coefficient of variation (CV%) distribution of metabolite intensities in QC samples; (**c**) time series plot of principal component 1 (PC1) of the QC samples after Pareto Scaling for PCA.

For each analysis method, five QC samples were inserted in the analysis queue, with equal intervals between the QC samples. Each method’s stability was evaluated based on the corresponding distribution of the QC samples in the PCA score plot, the time-series variation and the CV% distribution. A typical evaluation result is given in Figure 1, which was based on the metabolites detected in the first fraction of the hydrophilic extract. The QC samples clustered tightly (Figure 1a). For each analysis method, no conspicuous variance in the QC result was detected, even after 200 analyses (Figure 1c). Over 90% of the quantitation data had an RSD% below 20% (Figure 1b). This indicated that the adopted analysis strategies were acceptable. 

A total of 226 serum samples were subjected to metabolomics and lipidomics analyses. One result was unacceptable and was removed from the subsequent statistical analysis. After removing the missing values and those acquired from the QC samples with a CV% greater than 50%, 1171 compounds (Table S1) belonging to 30 chemical classes were identified (Figure S1).

**Figure 2:**
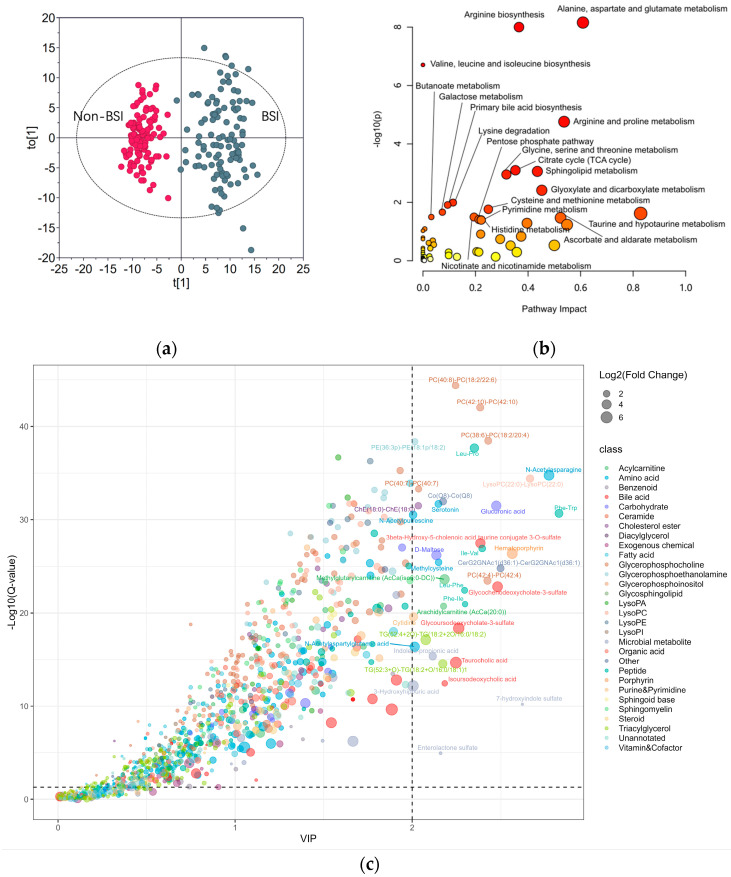
Exploring the BSI−affected metabolic pathways: (**a**) OPLS−DA score plot for BSI and nonBSI Samples; (**b**) metabolic pathway enrichment analysis highlighting the most perturbed pathways by infections; (**c**) bubble plot of metabolites with fold change > 2 and Q−value < 0.05 between BSI and non-BSI samples. VIP: variable importance.

The metabolomic data from the BSI and non-BSI patients were subjected to PCA. A clear separation between the two populations was discerned (Figure S2). To identify the potential perturbated metabolic pathways, a PLS-DA was conducted (Figure 2a). No overfitting was found in the separation of the two groups (Figure S3). Figure 2c shows the key metabolites contributing to the separation of the two populations. The pathway enrichment analysis indicated that the most perturbated metabolic pathways due to infection were the alanine, aspartate and glutamate metabolic pathways (Figure 2b). Arginine biosynthesis was also affected by the various microorganism infections.

### 3.2. Subgroup Differentiation of BSI Cases 

#### 3.2.1. Classification of the Pathogens into Four Groups

**Figure 3:**
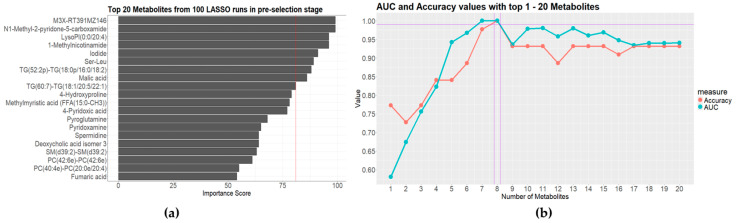
Results of the four-class separation of the isolated strains: (**a**) the best-performing model with top 20 metabolites from 100 LASSO runs in pre-selection stage; (**b**) AUC and accuracy values with top 1-20 metabolites in the evaluation stage.

Clinically, the empirical antibiotic administration strategies were different for the patients with Gram-positive and Gram-negative bacterial infections. This is also the case for bacterial and fungal infections. Thus, a primary pathogenic result could aid physicians in determining which types of microbes have infected their patients. To this end, we first tried to differentiate between Gram-positive bacterial, Gram-negative bacterial, fungal, and anaerobic bacterial infections among the BSI patients. A 3-Stage Recursive Feature Selection (3SRFS) process was conducted, employing different data analysis techniques to improve the stability of the selected metabolite panel. First, 76 metabolite variables were selected through a Sure Independence Screening (SIS) process using component-wise ANOVA. Then, a pre-selection procedure was conducted by imputing the 76 metabolites into three (computationally efficient) models (Least Absolute Shrinkage and Selection Operator (LASSO), Light Gradient Boosting Machine (LightGBM), and Random Forest (RF)) with different hyperparameters for feature ranking based on Permutation Feature Importance (PFI) and SHapley Additive exPlanations (SHAP). The top 20 features from each ranking were selected for further evaluation. Next, four classification models (LIghtGBM, RF, Support Vector Machine (SVM), and Extreme Gradient Boosting (XGBoost)) were trained separately with the top 20-feature panels to test the diagnostic performance, which was evaluated based on their classification accuracy and area under the receiver operating characteristic curve (AUC) values. The metabolites in the series with both accuracy and AUC values over a certain threshold (0.95) were considered potential candidates to be sent back to the pre-selection stage. This process was executed repeatedly until the result was sufficiently stable. The diagnostic metabolite panel with highest accuracy and AUC value consisted of the following metabolites: M3X-RT391MZ146, N1-Methyl-2-pyridone-5-carboxamide, LysoPI(0:0/20:4), 1-Methylnicotinamide, iodide, Ser-Leu, TG(52:2p)-TG(18:0p/16:0/18:2), and malic acid (Figure 3b).

#### 3.2.2. Differentiation Between Gram-Positive and Gram-Negative Bacteria

**Figure 4:**
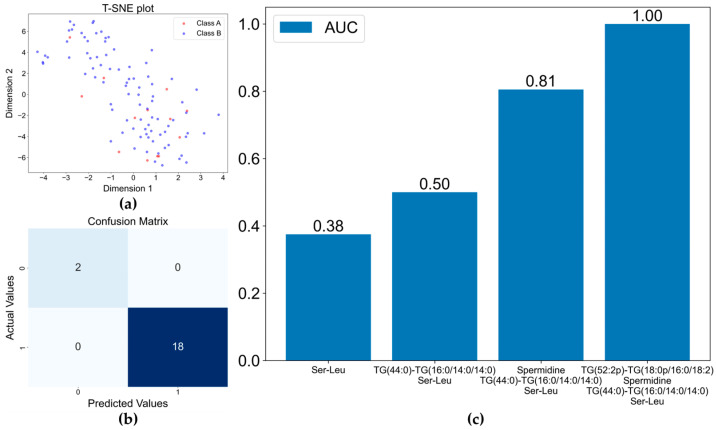
Separation of the Gram−positive and Gram-negative bacterial BSIs by XGBoost model: (**a**) t−SNE plot for classification of Gram−Positive and Gram−Negative bacteria; (**b**) confusion matrix of XGBoost model based on the four selected metabolite variables; (**c**) increase in AUC with the inclusion of more metabolites in the model.

Although the four microorganism groups could be differentiated to an acceptable standard, the most isolated pathogens from BSI patients in clinical practice are eubacteria [29]. Undoubtedly, the differentiation between Gram-positive and Gram-negative bacterial BSIs is helpful, at least for empirical antibiotic administration. To this end, we attempted to identify the differential metabolites between the BSIs caused by Gram-positive and Gram-negative bacteria. To more accurately and efficiently identify the differential metabolites, we generated a T-distributed Stochastic Neighbor Embedding (T-SNE) graph to obtain an overview of the collected data. The T-SNE graph showed no clear separation if all the detected metabolites were included (Figure 4a). This could be ascribed to the fact that the quantities of bacteria in the infected patients’ circulation were not comparable. Thus, the metabolites released from the bacteria in the blood varied significantly for different patients. In this setting, feature screening was necessary to identify the stable variables (metabolites) with higher feature importance. To this end, the metabolites detected in over 50% of the samples in each group were selected first. Then, the remaining variables with a maximum importance ≥ 0.005, calculated using the RF algorithm were also selected. The two sets of variables were combined, generating a dataset with 284 variables. Using the Recursive Feature Elimination algorithm, the XGBoost model was utilized to evaluate the 284 variables and carry out the differentiation. It was found that the two groups could be clearly differentiated (Figure 4b). The important metabolites for differentiating between Gram-positive and Gram-negative bacterial BSIs were spermidine, TG(44:0)-TG(16:0/14:0/14:0), Ser-Leu, and TG(52:2p)-TG(18:0p/16:0/18:2). Combining these four metabolites, complete differentiation could be realized (Figure 4c). Due to the similar molecular weights of TG(44:0) and TG(16:0/14:0/14:0), these two metabolites could not be individually identified. Thus, they were called TG(44:0)-TG(16:0/14:0/14:0). This was also the case for TG(52:2p)-TG(18:0p/16:0/18:2).

#### 3.2.3. Identification of *E. coli* and *K. pneumoniae* BSIs

**Figure 5:**
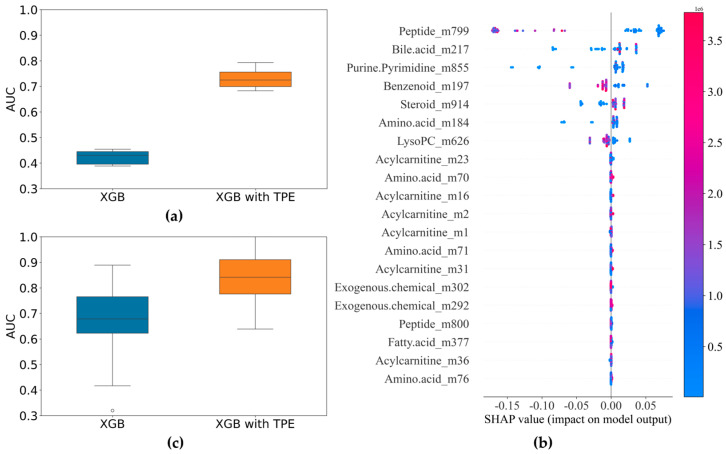
Results of the identification of *E. coli* and *K. pneumoniae* BSIs: (**a**) comparison of AUC before and after optimization of XGBoost model (87 variables); (**b**) SHAP summary plot of TPE−optimized XGBoost model showing metabolite contributions for bacterial differentiation; (**c**) comparison of AUC before and after optimization of XGBoost model (7 variables).

For bacterial BSIs, the most commonly isolated pathogenic bacteria are Gram-negative bacilli [30]. In our samples, the top two isolated species were *E. coli* and *K. pneumoniae.* Given that we could successfully differentiate between Gram-positive and Gram-negative bacterial BSIs, we tried to identify different Gram-negative bacillus species using the two *enterobacteria* species as examples. First, the 87 features selected through the SIS procession and correlation coefficient screening were incorporated into the XGBoost model and optimized using the Tree-structured Parzen Estimators (TPE) method. The AUC values before and after optimization are shown in Figure 5a. The TPE optimization improved the stability of the model classification predictions. The key features that contributed greatly to the classification model (identified using the SHAP method and the PFI methods) are ranked in Figure 5b. The top seven features were selected in turn and then subjected to modeling again using XGBoost. The median value of the AUCs in the XGBoost with TPE results was about 0.86, and the maximum value reached up to 1 (Figure 5c). The important metabolites for the differentiation of *E. coli* and *K. pneumoniae* BSIs were Ile-Gln/Leu-Gln, Glycocholic acid, N2, N2-Dimethylguanosine, Tyrosol 4-sulfate, 5alpha-Androstan-3beta,17alpha-diol disulfate, Tryptophan 2-C-mannoside, and LysoPC(0:0/22:5n3).

#### 3.2.4. Identification of ESBLs(−) and ESBLs(+) Cases

**Figure 6:**
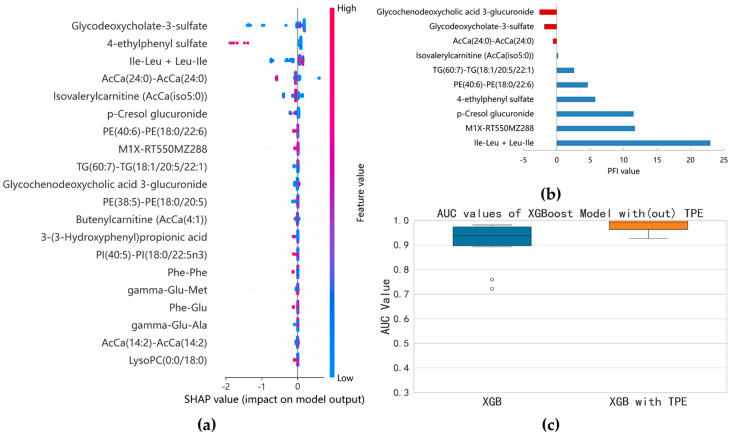
Results of the model separation according to ESBL−producing phenotypes: (**a**) SHAP summary plot of XGBoost model based on the optimal parameter combination; (**b**) top 10 features identified by permutation feature importance (PFI); (**c**) comparison of AUC before and after optimization of XGBoost model (six variables).

Since the advent of antibiotics, antibiotic-resistant bacteria have been constantly emerging. Bacteria have evolved many mechanisms to resist antibiotics, with the production of antibiotic hydrolysis enzymes being the most common strategy. ESBLs are enzymes that can hydrolyze antibiotics whose chemical structures contain beta-lactam rings. Currently, ESBL-producing bacteria are still an important public health concern. Bacteria with ESBL-producing abilities create more challenges in treating BSI patients [31]. Thus, we aimed to identify the bacteria with ESBL-producing abilities. Using the SHAP method, 71 features with non-zero SHAP values were selected. After comprehensively evaluating the key features using the SHAP and the PFI methods, the number of important variables decreased from 20 to 6 (Figure 6a,b). These variables were glycodeoxycholate-3-sulfate, PE(40:6)-PE(18:0/22:6), 4-ethylphenyl sulfate, p-cresol glucuronide, Ile-Leu/Leu-Ile, and an unannotated metabolite with an m/z ration of 228. The six metabolite variables were input into the XGBoost model again. The optimized model resulted in a stable AUC value near 1 with a median of 0.9907 (Figure 6c). 

## 4. Discussion

Once a host is invaded by a certain pathogen, the host must respond to the invasion. This defense mechanism is linked to metabolic reprogramming, which involves numerous cell types and metabolic pathways [32]. In addition, the BSI pathogen is metabolically bioactivated, so the host’s serum metabolome is perturbed. 

In recent years, significant efforts have been made to improve the strategies for high-dimensional-data-based multi-group classification [33,34,35,36]. Mainly motivated by the work of Fan et al. [36], a novel multi-step feature selection method called 3RHFS was first introduced in this study. This method was used because the amount of a specific pathogen in the circulation differs between BSI patients. The exact number of clone-forming units (CFUs) of a pathogen in a blood sample is affected by the sampling time, sampling strategies, and the patient’s immunity [37]. This variability in the number of CFUs results in varying degrees of detectability of pathogen-secreted and host-response metabolites in the serum. To solve this problem, we introduced a microbial metabolomic data processing strategy (3RHFS) to address the uncertainties in the metabolites from both the host and pathogens. Through 3RHFS, we achieved successful differentiation of the four major pathogenic microorganism groups (Figure 3). 

Another contribution of this work was the identification of important metabolites under different circumstances. It was challenging to identify the small subset of key variables that contributed the most to the subgroup differentiation out of the 1171 detected metabolites, especially since the number of variables was much higher than the number of observations and because the important variables were highly correlated with some unimportant ones. In this setting, the adoption of the SIS method could easily remove the features that were most likely to be dependent on certain labels and irrelevant for classification purposes. If two variables were highly correlated, they would show similar variation trends and might carry identical information. The inclusion of such redundant variables could negatively impact the model’s performance. To solve this problem, we calculated the correlation coefficients between metabolite variables. The selected metabolites must be involved in the interactions between the host and the pathogens. After acquiring the modeling results, we further checked the contribution of the metabolites to confirm that they were important in distinguishing between different BSI cases. Using this strategy, the two difficult problems of differentiating between the four major pathogen groups and the two biologically similar *enterobacteria* were solved satisfactorily (Figure 3 and Figure 5).

Correctly distinguishing BSI patients from non-BSI patients is crucial in guiding clinical decision-making. Serum metabolome changes are useful indicators for the occurrence of BSIs. From this study, it was clear that the BSI’s effects on the serum metabolome could be largely ascribed to perturbations to amino acid metabolism (Figure 1). It was reported that the amino acids involved in the urea cycle are dysregulated in infectious diseases [38]. In this study, metabolites from arginine, aspartate, and glutamate metabolism greatly contributed to differentiating BSIs from non-BSIs (Figure 1c). Tricarboxylic acid (TCA) cycle metabolism was also affected by BSI (Figure 1c). It was reported that the TCA cycle is crucial in maintaining pro-/anti-inflammatory homeostasis [39]. Sphingolipid metabolism also played a key role in the differentiation between BSIs and non-BSIs. Many sphingolipid signaling pathways modulate infection and immunity, and sphingolipid metabolism could be affected differentially by different bacterial genera [40,41]. Glyoxylate is only metabolized by bacteria, and the activity of glyoxylate and dicarboxylate metabolism is affected by the supply of oxygen [42]. Presumably, the contribution of glyoxylate metabolism might arise from the anaerobic bacteria, but this conclusion should be confirmed by an expanded number of anaerobic bacterial BSI cases. Collectively, the metabolic perturbations caused by BSIs are explainable based on the relevant metabolic pathways’ biological roles.

The isolated pathogens in this study could be roughly classified into four groups. These were Gram-positive bacteria, Gram-negative bacteria, fungi, and anaerobic bacteria. This classification was based on their pathogenic characteristics, empirical antibiotic selection strategies, and cell wall structures. If a patient was judged to be suffering from a BSI (Figure 2a), classification of the pathogenic groups (Figure 3) could guide empirical antibiotic administration. Nearly 20 metabolites were needed to achieve a satisfactory classification of the four pathogenic groups (Figure 3). The metabolites that contributed to the differentiation included different kinds of chemicals, indicating the need for different parameters of various aspects. Of note, there was a relatively limited number of fungal and anaerobic bacterial isolates. In the future, the model should be tested using a larger sample size with expanded fungal and anaerobic bacterial species. 

After successfully separating the four major pathogenic groups, we focused on differentiating between the isolated Gram-positive coccus and Gram-negative bacillus pathogens. These two classes of bacteria are involved in over 80% of the BSIs [43]. In theory, the separation of two groups is easier than separating four groups. This conclusion was proved by the results shown in Figure 4. Using only four metabolites was sufficient to distinguish between the Gram-positive coccus and Gram-negative bacillus bacteria. Two triglycerides appeared to be the key metabolites for the separation. Two triglycerides appeared to be the key metabolites for the differentiation and are absent in the data shown in both Figure 2 and Figure 3. A previous analysis reported that BSIs caused by Gram-positive strains are characterized by specifically fluctuated triglycerides, which are not found in Gram-negative infections [44].

Nearly all the isolated Gram-negative bacteria were *E. coli* and *K. pneumoniae*, which are also the two most commonly isolated strains from BSIs in clinical practice [43]. We tried to identify them by using the collected metabolomics data, with encouraging results (Figure 5). Notably, some of the metabolites that play key roles in differentiating between the two bacteria are bacteria-specific metabolites and are co-metabolized by the host and bacteria.

For BSIs caused by *E. coli* and *K. pneumoniae*, the most important issue was to ascertain if the isolates were ESBL-producing strains [45]. These strains are usually resistant to many classes of antibiotics, and BSIs with these strains are more lethal to patients. Therefore, we tried to identify the strains with ESBL-producing abilities. Fortunately, the ESBL-positive and ESBL-negative phenotypes could be correctly identified (Figure 6). Unfortunately, these metabolites were not identified.

Although we successfully differentiated the targeted microorganisms, there were limitations to this study. First, the number of each studied strain was not high enough. For some species, there were too few isolates to be statistically analyzed in the current study. Second, there were some metabolites that could not be identified. This could influence the potential infection-related mechanism that was proposed. Lastly, it was difficult to quantify the exact number of CFUs of the pathogenic organisms in the blood. Thus, the limit of detection for the different BSI samples could not be identified. Our future studies will address these topics.

## 5. Conclusions

When patients are infected by pathogens, both the host and microorganisms will respond to the infection. As long as there are living microorganisms in the bloodstream, they will release their metabolites into the blood. The host will also react to the infection and induce anti-infection immunity. This will reprogram the host’s normal metabolism, resulting in metabolome alterations. Through a metabolomic analysis, we were able to differentiate between BSIs and non-BSIs. In addition, the isolated strains were differentiated based on their genus, family, cell wall structure, and antibiotic-resistant phenotypes. The merit of this metabolomic analysis was that it is independent of the availability of BC results and requires less time. Compared to the mNGS strategy, metabolomic analyses are less expensive and easy to apply. For multi-group separation, differentiating between strains within the same family, or to show close relationships, we proposed two practical data processing methods. If the data size can be expanded to include as many pathogens as possible, the constructed separation models could be tested for clinical utilization. In summary, the proposed strategy could detect BSIs using blood samples at an early stage of infection. 

## Data Availability

The raw metabolomics data and the 3RHFS algorithm program are available on reasonable request.

## Supplementary Materials

The following supporting information can be downloaded at https://www.mdpi.com/article/10.3390/metabo14120685/s1: Figure S1: Pie chart of metabolite chemical classes structurally annotated in this experiment; Figure S2: Unsupervised PCA score plots of metabolomic phenotypes between non-BSIs (control) and BSIs (A, B, C, and D represented the four major groups in Figure 3). The metabolomic data were log-transformed and center-scaled for modeling. Model parameter: R 2 X = 0.309 (cumulative variance proportion of two principal components); Figure S3: 999 times permutation to test robustness of OPLS-DA modeling differentiating BSIs vs. non-BSIs.
