# Supplementary material for: Early Diagnosis of Bloodstream Infections Using Serum Metabolomic Analysis
Source: Metabolites. 2024 Dec 6;14(12):685. doi: 10.3390/metabo14120685 (PMC11676852; doi:10.3390/metabo14120685)
Supplement: Supplementary file 1 [file metabolites-14-00685-s001.zip › metabolites-3297116-supplementary/metabolites-3297116-supplementary.pdf]

Supplymentary materials and methods

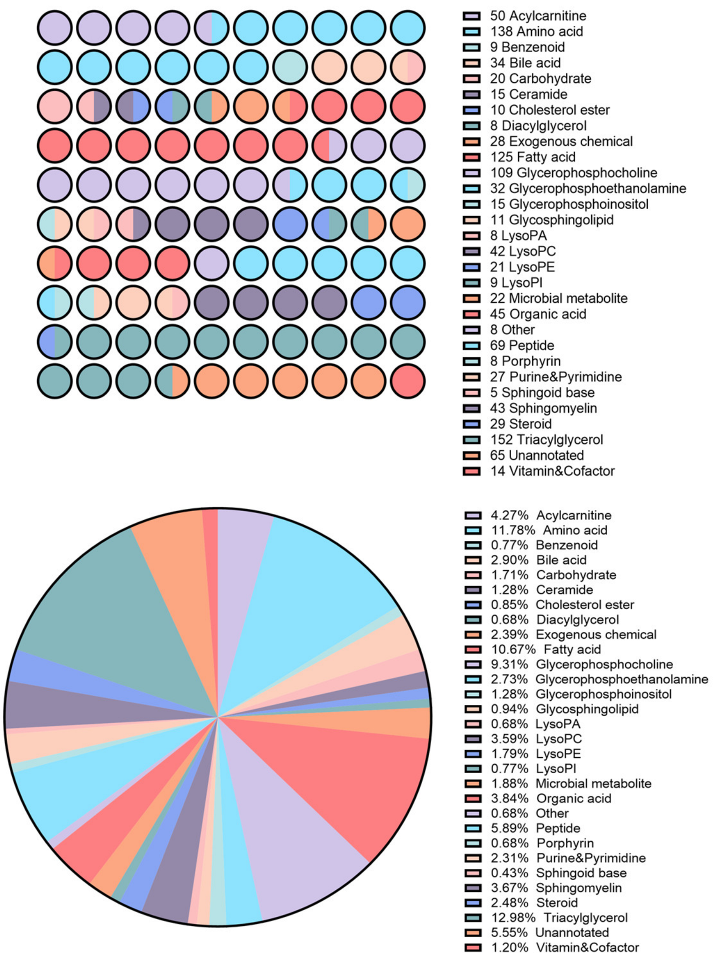

Figure S1 The identified metabolites classified according to their chemical structures in this study.

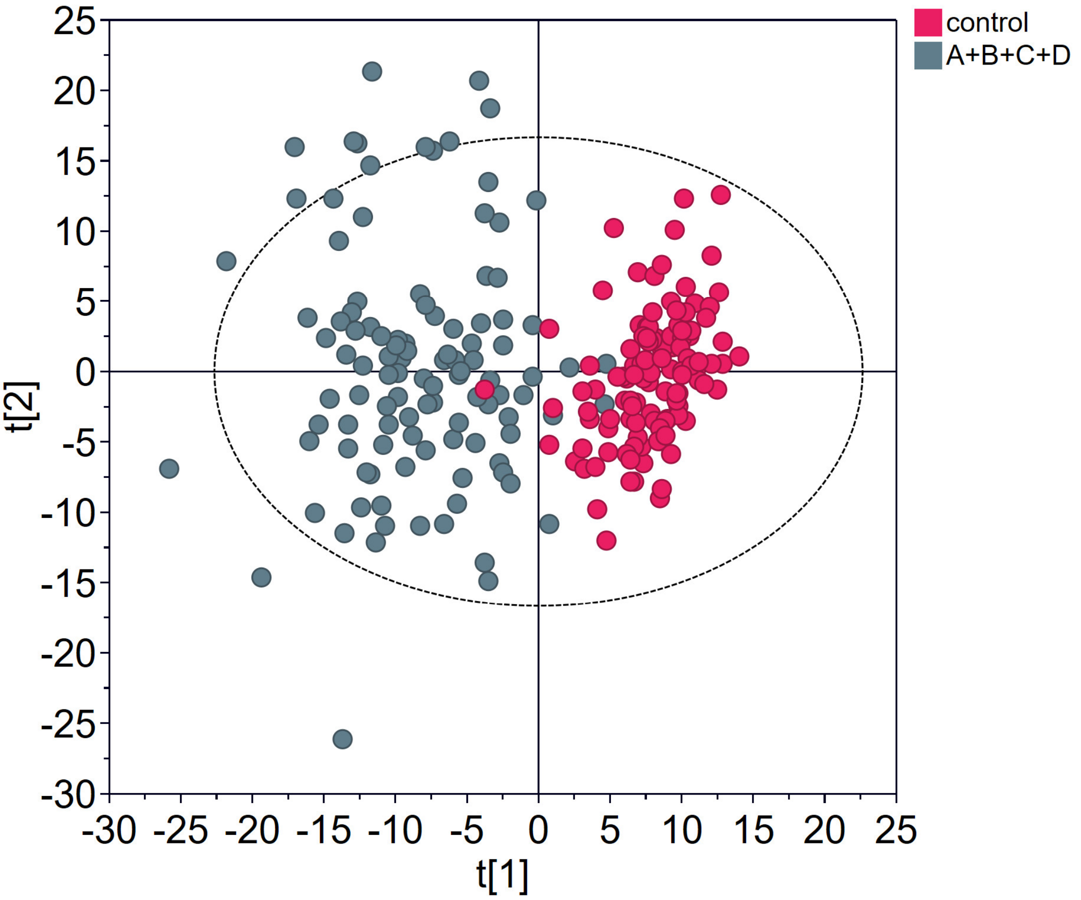

Figure S2 PCA results of the separation between BSI group (A+B+C+D) and the non-BSI group (control) using the metabolomics data.

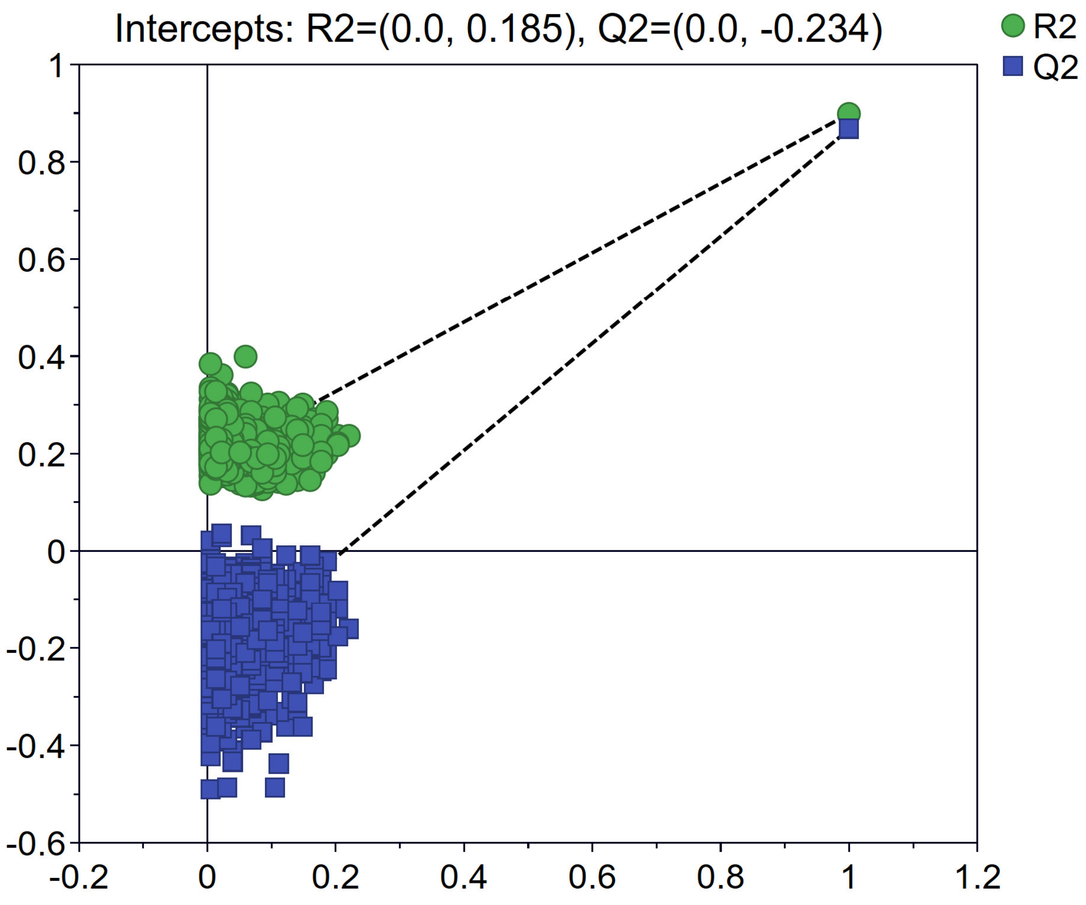

Figure S3 The permutation test parameters showed no overfitting of the PLS-DA results.
